# Weight Management in Young Adults: Systematic Review of Electronic Health Intervention Components and Outcomes

**DOI:** 10.2196/10265

**Published:** 2019-02-06

**Authors:** Taylor Jade Willmott, Bo Pang, Sharyn Rundle-Thiele, Abi Badejo

**Affiliations:** 1 Social Marketing @ Griffith Griffith Business School Griffith University Nathan Australia

**Keywords:** body weight maintenance, eHealth, health behavior, obesity, overweight, review, technology, weight gain, young adult

## Abstract

**Background:**

Young adulthood is a vulnerable period for unhealthy lifestyle adoption and excess weight gain. Scant attention has been focused on developing and evaluating effective weight gain prevention strategies for this age group. Electronic health (eHealth) offers potential as a cost-effective means of delivering convenient, individually-tailored, and contextually-meaningful interventions at scale.

**Objective:**

The primary aim of this systematic review was to locate and synthesize the evidence on eHealth weight management interventions targeting young adults, with a particular focus on (eHealth) intervention components and outcomes.

**Methods:**

A systematic review was conducted in accordance with the Preferred Reporting Items for Systematic Reviews and Meta-Analyses guidelines. The search strategy was executed across the following electronic databases: Cumulative Index to Nursing and Allied Health Literature, Cochrane Library, EBSCO, EMBASE, Emerald, Education Resources Information Center, Medical Literature Analysis and Retrieval System Online, Ovid, ProQuest, PsycINFO, PubMed, Science Direct, Scopus, and Web of Science. Furthermore, 2 reviewers independently assessed records for eligibility: peer-reviewed, published in English, and report evaluations of eHealth weight management interventions targeting healthy young adults (aged 18-35 years). Data were then extracted from studies that met the criteria for inclusion. The methodological quality of studies was independently assessed by 2 reviewers using the Effective Public Health Practice Project’s (EPHPP) quality assessment tool. A comprehensive narrative evidence synthesis was then completed.

**Results:**

Out of the 1301 studies assessed for eligibility, 24 met the criteria for inclusion. According to the EPHPP quality assessment tool, overall, 19 studies were as rated weak, 5 as moderate, and none as strong. The narrative synthesis of intervention outcomes found 8 studies reported positive weight-related outcomes, 4 reported mixed outcomes, and 12 did not report any significant changes in weight-related outcomes. The narrative synthesis of (eHealth) intervention components led to 3 levels of classification. A total of 14 studies were classified as *Web-based*, 3 as *mobile-based*, and 7 as *multicomponent* interventions. Following the narrative synthesis, 5 key strategies were thematically identified: self-regulation (goal setting and self-monitoring), tailored or personalized feedback, contact with an interventionist, social support, and behavioral prompts (nudges and reminders) and booster messages.

**Conclusions:**

Findings highlight the limited evidence base for eHealth weight management interventions targeting young adults. The complex nature of weight management presents an ongoing challenge for interventionists to identify *what works, for whom, how, and when*. The quality of the evidence in this review was generally assessed as weak; however, assessment tools such as the EPHPP are principally concerned with *what should be* and this is seldom equivalent to *what works*. Thus, while sampling, study design and retention rates will remain key determining factors of reliability and validity, further research attention directed toward the development of guiding tools for community trials is warranted.

## Introduction

### Background

Nearly one-third of the global population is overweight or obese, that is, more than 2.1 billion people [1]. The prevalence of obesity is rising rapidly throughout both the developed and the developing world, creating a substantial social, economic, and health burden on society [2]. If current trends continue, it is estimated that by 2030 almost half of the world’s adult population will be overweight or obese [3]. Such a scenario would have devastating consequences for the global burden of noncommunicable diseases, with increasing body mass index (BMI) associated with an elevated risk of developing a chronic disease such as cardiovascular disease, diabetes, respiratory disease, and certain cancers [4]. The magnitude of the obesity epidemic has led to a shift in focus from the clinical treatment of obesity to the development of prevention strategies that address the economic, environmental, sociocultural, and lifestyle-related causes of population weight gain [5-7]. The prevention of weight gain and the maintenance of a healthy weight are considered less challenging, less expensive, and potentially more effective than the treatment of obesity after it has fully developed [8]. Once established, obesity is difficult and costly to treat [9,10]. Owing to the projected increases in obesity prevalence, the challenges faced in delivering effective treatment, and the costs associated with treatment, it will not be possible to deliver care for all individuals in need [11]. Therefore, the prevention of obesity and its comorbidities are, and must continue to be, a foremost public health priority.

Targeting high-risk groups with prevention interventions is hypothesized to have the greatest impact on the rising incidence of overweight and obesity [4]. Efforts to prevent obesity have mainly focused on children and adolescents, whereas other important age groups have been overlooked [12]. The most rapid weight gain in the life course has been observed during the early twenties to midthirties [12,13], with incident obesity at a younger age associated with increased risk of chronic disease and mortality in later adult life [12,14,15].

Young adulthood is a transitional life stage in which young people experience significant life changes, increasing independence, and adopt lasting health behavior patterns [16]. The cause of age-related weight gain during young adulthood appears to be lifestyle-based, resulting from marked declines in physical activity (PA), increases in sedentary behavior, and poor dietary habits [17-22]. These changes in PA and diet-related behaviors likely result from the significant life transitions that occur during young adulthood, such as moving out of the family home, relocating to new environments, beginning full-time work or tertiary study, and establishing financial, residential, and employment stability [16]. Among this demographic, barriers to healthy weight maintenance exceed enablers [23], with healthful eating and regular PA not considered high priorities [24]. Perceived time constraints, lack of discipline, inadequate self-regulation skills, and a lack of environmental support for healthy eating and PA have all been cited as common barriers to healthy weight maintenance among young adults [23-26]. Common enablers to healthy weight maintenance include education and awareness (eg, what to eat and what not to eat), self-regulation skills (eg, practicing moderation and portion control), and positive social and environmental support [23,24,26]. Importantly, the adoption of healthier lifestyle behaviors in young adulthood has been associated with a lower risk of developing chronic disease in later adult life [27]. Given obesity is entirely preventable, the establishment and maintenance of healthy behavioral patterns in young adulthood would deliver long-term health benefits to individuals as well as cost benefits to society. Therefore, a more fine-grained understanding of the means that can be reliably used to effectively assist young adults in managing their weight is needed.

### Review Rationale and Aim

Previous reviews [28-33] of lifestyle interventions for obesity prevention and weight management have highlighted the limited evidence base for successful interventions among this age group. Findings from these reviews were inconclusive owing to the small number of studies available [30], small sample sizes [30,32], heterogeneity across intervention designs [30,31], differences in participant characteristics [30], gender biases [32], and short intervention durations [30,32]. Traditional weight management interventions (ie, face-to-face sessions with a trained interventionist) may not meet the needs of many young adults, as evidenced by lower recruitment and retention rates, inferior attendance and compliance, and poor weight-related outcomes relative to older adult participants [34]. Traditional interventions are resource intensive in terms of the commitment required by participants and intervention providers, which can create barriers for full participation and adherence [29]. Moreover, the resources required to deliver face-to-face interventions (individual or group-based) prevent large-scale deployment to the wider community [28].

Young adulthood is a developmentally unique life stage [16]. Therefore, weight management interventions aimed at this demographic must have a specific focus on the distinct challenges faced by young adults that are known to contribute to weight gain, including rapidly shifting life circumstances related to home, work, family, and other relationships [16]; examples of the challenges faced during this developmental period include juggling the many responsibilities that come with being an ‘adult’ [16], continuing cognitive development through the midtwenties (eg, impulse control, regulation of emotions, and rational decision making) [35], and learning the skills needed to sustain oneself, such as home food preparation and meal planning [36,37]. Technology may offer a cost-effective means of engaging young adults in weight management, with the current generation of young adults among the highest users of digital technologies such as social media, mobile phones, and wireless information sharing platforms [38].

Electronic health (eHealth), defined as the use of information and communication technologies (ICTs) for health [39], offers a feasible alternative to traditional weight management interventions and has the potential to be delivered at scale. Telemedicine, first used in the 1920s, is the oldest form of eHealth. The introduction of broadband internet in the 1990s, followed by wireless technologies, precipitated an explosion of eHealth and mobile health apps within the health care field [40]. Interventions that encompass ICTs (eg, internet-enabled mobile and tablet devices, wearable monitors) permit the efficient delivery of individually-tailored, context-specific health behavior change programs, with time-unlimited feedback, coaching, and support [41]. The popularity, mobility, and capability of modern ICTs allow temporal synchronization of intervention delivery and allow the intervention to be delivered at a convenient time and place [41]. For example, young adults may be sent a short message service (SMS) text message in the morning to remind them that having a nutritious breakfast is important for healthy weight maintenance [42], with a link to healthy breakfast recipes based on items commonly available at home. eHealth-based interventions have previously demonstrated the potential to promote healthful changes in both diet and PA behaviors [43] and have been used as a treatment option for obesity in adults [44]. However, there is limited evidence on the effectiveness of eHealth-based approaches for weight loss maintenance and weight gain prevention [44], especially among young adult populations. As such, the primary aim of this review was to locate and synthesize the evidence on eHealth weight management interventions targeting young adults, with a particular focus on (eHealth) intervention components and outcomes.

## Methods

### Review Protocol

This systematic review was conducted in accordance with the Preferred Reporting Items for Systematic Reviews and Meta-Analyses (PRISMA) guidelines [45]. Refer to Multimedia Appendix 1 for the PRISMA checklist used in this review.

### Data Sources and Search Methods

The systematic literature search was completed in September 2018. The search strategy was executed across the following electronic databases: Cumulative Index to Nursing and Allied Health Literature, Cochrane Library, EBSCO, EMBASE, Emerald, Education Resources Information Center, Medical Literature Analysis and Retrieval System Online, Ovid, ProQuest, PsycINFO, PubMed, Science Direct, Scopus, and Web of Science. As outlined by Smith et al (2011) in a systematic review of individual studies, the search should be as wide as possible to maximize the likelihood of capturing all relevant data and minimizing the effects of reporting biases. As such, a search of a wide variety of electronic databases relevant to the topic of interest is recommended as a best practice [46]. The predetermined search strategy was designed by combining relevant search terms related to eHealth, weight management, and young adults. Search terms were divided into 4 groups: (1) intervention type (ie, eHealth variations), (2) outcome (ie, weight-related and behavioral variations), (3) study design (ie, study type variations), and (4) participants (ie, young adult variations). The full search strategy and database results are provided in Multimedia Appendix 2. The reference lists of all included papers (backward search) and pertinent systematic reviews [28-33] were also hand searched to identify additional studies for inclusion. Google Scholar was used to screen papers citing included studies (forward search).

### Study Screening and Selection

All records were downloaded to Endnote Version X8 (Clarivate Analytics), duplicates were removed, and the remaining studies were assessed for eligibility via title and abstract by 2 independent reviewers. The results were categorized by title and abstract into (1) papers appearing to meet study selection criteria, (2) papers that should be retrieved for further examination, and (3) excluded papers. In cases where there were several publications from the same cohort, the study with the longest follow-up was selected; if the follow-up was equivalent, the most recent study was included. The full-text of potentially relevant papers was then obtained and assessed by 2 independent reviewers. These papers were further categorized. At all stages, any discrepancies were discussed and resolved by consensus. Where consensus could not be reached, a third independent reviewer acting as an arbitrator was consulted.

### Eligibility Criteria

The eligibility criteria adopted in the present review are as follows. To be included in the review, studies had to (1) be peer-reviewed, (2) be published in the English language, (3) report evaluations of eHealth weight management interventions targeting young adults (aged 18-35 years old), including randomized controlled trials (RCTs), controlled clinical trials (CCTs), and cohort studies (pretest-posttest and posttest only), (4) include participants who were healthy and free of acute illness or chronic disease, and (5) report a measure of weight pre and postintervention.

For the purposes of this review, eHealth referred to behavior change interventions, which were operationalized and transformed for delivery via ICTs including computers, tablets, mobile phones, wearable and nonwearable tracking devices, and digital games. For studies to be eligible for review, eHealth had to form the primary means of intervention delivery in *at least one* treatment arm. The technology could be used as both a tool to enable a process, function or service, or as the embodiment of eHealth itself [47]. Weight management was defined as the prevention of weight gain via the maintenance of a healthy body weight or the reversal of small gains to maintain a healthy body weight [8]. Studies that purposively recruited and subsequently evaluated weight loss or weight loss maintenance interventions among the obese (mean BMI>30 kg/m^2^) were excluded as the prevention of weight gain (ie, management) was the focus of this study; participants who have lost a significant amount of weight do not represent the general young adult population [48]. The age range of 18 to 35 years was selected based on the protocol included in the *National Heart, Lung, and Blood Institute’s* Early Adult Reduction of Weight through Lifestyle Intervention trials [49]. Weight gain is most rapid during these years [12,13], and increasing BMI in young adulthood increases the risk of developing metabolic syndrome over the subsequent 15 years almost 20-fold [50].

**Figure 1:**
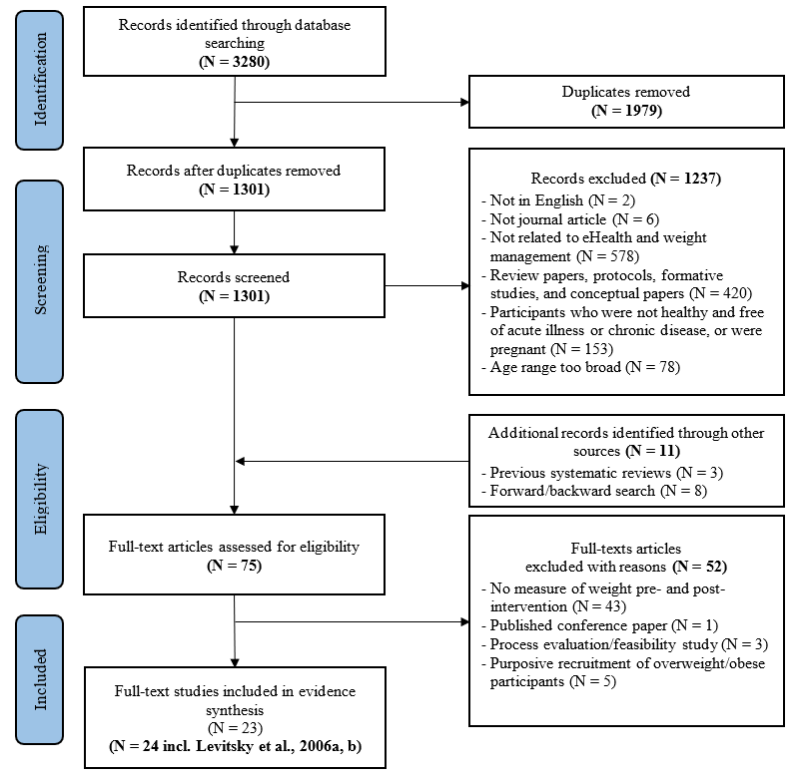
Preferred Reporting Items for Systematic Reviews and Meta-Analyses flowchart of study selection process.

Studies were excluded on the basis of the following criteria: (1) not peer-reviewed, (2) not in English, (3) not related to eHealth *and* weight management, (4) not an intervention evaluation, (5) included participants who were not healthy and free from acute and chronic disease, or were pregnant, (6) did not report a measure of weight pre and postintervention, or (7) did not specifically target young adults (aged 18-35 years). Studies that did not report an age range, the mean age of the sample, or the percentage of the sample who were within a given age range, were also excluded. Numbers and reasons for exclusions are reported in Figure 1.

### Data Extraction and Management

A data extraction form informed by the PRISMA statement was developed for abstracting study characteristics [45]. Data extracted included the following: study details (author, year of publication, and country), study design, participants (sample size, characteristics, setting, retention, and blinding), intervention and comparator details, duration, and data collection methods, measures, outcomes, and conclusions (refer to Multimedia Appendix 3). Following this, summary tables were thoroughly and independently reviewed by all authors for accuracy and relevance. Any inconsistencies were resolved through discussion.

### Quality Assessment

The Effective Public Health Practice Project’s (EPHPP) quality assessment tool [51] for quantitative studies was used to assess the methodological quality of included studies. The tool requires the assessment of 6 individual quality components (selection bias, study design, confounders, blinding, data collection methods, and withdrawals and dropouts) before assigning an overall quality rating (strong, moderate, or weak) based on a 3-point scale. The tool has been judged suitable for use in systematic reviews of effectiveness [52] and has been reported to have content and construct validity [51,53]. Moreover, a study comparing the EPHPP quality assessment tool with the Cochrane Collaboration Risk of Bias (CCRB) tool found the EPHPP tool to have fair interrater agreement for individual domains and excellent agreement for the final grade. In contrast, the CCRB tool had only slight interrater agreement for individual domains and fair interrater agreement for the final grade. Of note, no agreement between the 2 tools was evident in their final grade assigned to each study. The authors concluded that although both tools were developed to assess *quality of the evidence*, they appear to measure different constructs [54]. In the present review, 2 independent reviewers completed assessments of methodological quality according to the EPHPP tool. Any discrepancies were resolved through discussion with a third independent reviewer, acting as an arbitrator, when required.

### Data Synthesis

In line with the primary aim of this review, a comprehensive narrative evidence synthesis was completed. Each study was of intrinsic interest on its own and combining such complex interventions was likely to yield a meaningless result that would not provide actionable insights for improving the design of future interventions [55]. As such, the reviewers sought to describe the variation in study findings by qualitatively examining (eHealth) intervention components and outcomes rather than attempting to combine findings into 1 overall estimate of effectiveness [51].

Studies were categorized into 3 groups based on (eHealth) intervention components: *Web*-based, *mobile*-based, and *multi* component. Web-based refers to interventions that were predominantly delivered through the use of internet-enabled functions such as e-learning platforms, websites, and email. Mobile-based denotes interventions that were primarily delivered through mobile-enabled functions including SMS text messages and mobile phone apps. Multicomponent represents interventions that used a combination of the above technologies to deliver the intervention. Behavioral change strategies were thematically identified using the Coventry, Aberdeen, and London-Refined taxonomy [56].

Outcomes were classified as *positive* if there was a significant desired change in the weight-related measure postintervention delivery, for example, decrease or maintenance in body weight, BMI, or %body fat. Outcomes were classified as *mixed* if not *all* weight-related changes were statistically significant in *all* intervention arms. Outcomes were classified as having *no change* if no statistically significant differences in the weight-related measure were reported postintervention (*and* when compared with control groups, if applicable).

## Results

### Search Results

The initial database search located 3280 records, 1979 duplicates were removed, and the remaining 1301 records were retained for title and abstract screening. Of these, 1237 were excluded as they did not meet the criteria for inclusion. The most common reasons for exclusion were the following: (1) not related to eHealth and weight management, (2) not an intervention, (3) participants had an acute illness or chronic disease, or were pregnant, or (4) the age range was too broad (eg, 18-65 years). A total of 75 full-text papers were retained and assessed for inclusion. Of these, 24 studies met the criteria for inclusion and were included in the narrative evidence synthesis. Figure 1 illustrates the PRISMA study selection process employed.

### Study Characteristics

Of the 24 studies included, over 92% (n=22) were published from 2010 onward, and all were conducted in developed countries: a total of 17 in the United Sates [57-72], 4 in Australia [73-76], 2 in the United Kingdom [77,78], and 1 in Belgium [79]. The majority employed either a CCT design (n=7) or an RCT design (n=15). All interventions addressed weight management; however, the behavioral focus of each intervention differed: a total of 10 focused on both healthy eating and PA [61,62,64-67,69,71,73,74], 7 focused on multiple behaviors (eg, healthy eating, PA, stress management, and sleep) [57,59,60,72,76-78], 3 focused on self-weighing [63,70], 3 focused on PA only [58,68,79], and 1 focused on healthy eating only [75]. The number of participants within each of the studies ranged from 12 to 2621, with a mean sample size of 468 participants. The majority of studies (n=20) recruited participants from colleges or universities, with only 4 studies extending their recruitment beyond an academic setting [74-76,79]. The duration of interventions ranged from 6 weeks to 24 months (mean=22 weeks), with an average retention rate at the final point of data collection of 79%. In terms of outcomes, 12 out of the 24 studies did not report any statistically significant changes in the weight-related measure(s) [57,58,61,65,66,68,71-73,77-79], 8 reported significant positive weight-related changes (eg, maintenance of a healthy weight or reversal of small gains) [60,62-64,70,74,76], and 4 reported mixed outcomes [59,67,69,75]. Refer to Multimedia Appendix 3 for a summary of individual study characteristics.

### Intervention (Electronic Health) Components

Of the 24 studies included this review, 14 evaluated Web-based interventions [59-70,78], 3 evaluated mobile-based interventions [58,75,79], and 7 evaluated multicomponent interventions [57,71-74,76,77]. The following section provides a narrative synthesis of the different eHealth components employed in these studies.

#### Web-Based Interventions

Among the 14 studies evaluating Web-based interventions, 4 [65-67,78] comprised a Web-based education (e-learning) program, 8 [59-64,68,69] used a combination of internet-enabled functions (eg, e-learning, website, email, e-counselor, e-newsletter, and Wi-Fi enabled scale), and 2 [70] used email as the sole method of intervention delivery. Typically, in the e-learning-based programs, participants (college or university students) were required to enroll in the program and complete the required modules to receive course credit for their participation [59,64,65,67,69,72]. The main behavioral change strategies employed in the e-learning-based studies were knowledge shaping, self-monitoring, social support, and contact with an interventionist (see Multimedia Appendix 3). For instance, in the study conducted by Gow et al (2010), students were randomized to either the (1) *internet* intervention arm (6 intensive e-learning sessions delivered via Blackboard), (2) *feedback* intervention arm (encompassing feedback from interventionists and using Blackboard for self-weighing) or (3) the *combined* intervention arm (e-learning sessions plus feedback) [69]. Similar programs were designed and evaluated by Dennis et al (2012), Greene et al (2012), LaChausse et al (2012), Kattelmann et al (2014), and Nikolaou et al (2015), whereby students randomized to the intervention arm(s) completed a semester long e-learning program accessible via a centralized website [57,65-67]. Specifically, Nikolaou et al (2015) used the Web-based e-learning program *Moodle* to deliver intervention content, with weekly email reminders sent to alert participants of new materials and mailboxes used to encourage communication between participants and interventionists [62]. Conversely, the study conducted by Harvey-Berino et al (2012) used a Web-based e-learning platform to facilitate weekly Web-based synchronous *group chats* led by a trained interventionist and supported by materials accessible via the intervention website [64].

The remaining Web-based interventions integrated multiple internet-enabled functions including e-newsletters, social network sites (SNSs), and email. The main behavioral change strategies employed in these studies were knowledge shaping, goal setting, self-monitoring, and contact with an interventionist (see Multimedia Appendix 3). For example, the study conducted by West et al (2016) was delivered via weekly e-newsletters and a private Facebook page, with participants also receiving a Wi-Fi scale and PA tracker (Fitbit Zip) for self-monitoring [72]. Similarly, the study conducted by Schweitzer et al (2016) comprised an adapted eHealth intervention where participants received weekly emails with tips for achieving set goals and weblinks to their personal accounts for viewing progress and accessing additional material [61]. In contrast, Bertz et al (2015) used Wi-Fi scales and email to facilitate the implementation of the caloric titration method, which involves daily self-weighing and visual feedback to promote weight management [63]. The final 2 studies combined both Web-based and offline components. The intervention evaluated by Dennis et al (2012) integrated both Web-based modules and biweekly in-class sessions with an expert instructor in nutrition and exercise science [67]. Similarly, the *Choosing Healthy Options in College Environments and Settings* (CHOICES) trial evaluated by Lytle et al (2017) offered an academic course with e-learning, face-to-face, and hybrid options for program delivery. The Web-based program included e-learning modules, an SNS, and a support website [59].

#### Mobile-Based Interventions

The 3 studies evaluating mobile-based interventions [58,75,79] delivered intervention content primarily via SMS text messages and mobile phone apps. The main behavioral change strategies employed in the mobile-based studies were goal setting, self-monitoring, and behavioral prompts (see Multimedia Appendix 3). For example, in the study conducted by Munoz et al (2014), participants used a pedometer to track PA, with brief SMS text messages (2-3 per week) sent throughout the intervention period to encourage the adoption of healthy behaviors [58]. Similarly, in the study conducted by Kerr et al (2016), dietary intake was monitored using a mobile food record app and tailored dietary feedback was sent weekly via SMS text messages to nudge healthy eating habits [75]. The study conducted by Simons et al (2018) comprised an investigator-designed mobile phone app (Active Coach) and a wearable device (Fitbit Charge) for tracking PA. The app included goal-setting functionalities, practical tips, and educational facts [79].

#### Multicomponent Interventions

The 7 studies [57,71-74,76,77] that were categorized as multicomponent used various eHealth technologies to deliver or support the intervention. The multicomponent studies employed a larger number of behavioral change strategies including knowledge shaping, barrier identification, goal setting, outcome expectation setting, behavioral prompts, self-monitoring, graded tasks, skill development, personalized feedback, social support, and contact with an interventionist (see Multimedia Appendix 3). The more complex interventions such as *TXT2BFiT* [73,74] and *HEYMAN* [76] incorporated multiple (eHealth) intervention components and associated change strategies. The pilot TXT2BFiT study comprised short SMS text messages, emails, mobile phone apps, and an internet forum [73]. The pilot was later refined and trialed in a larger RCT, which encompassed coaching calls by a dietician skilled in motivational interviewing, personalized SMS text messages tailored to participants’ stage of change to prompt behavior change, a website (resource bank) for knowledge shaping, and 4 designer mobile phone apps for goal setting and self-monitoring. Following the completion of the 12-week intervention, booster SMS text messages, emails, and coaching calls were used to promote long-term behavioral change [74]. Similarly, the HEYMAN study included a website (resource bank) for knowledge shaping, wearable PA tracker (Jawbone) for goal setting and self-monitoring, weekly face-to-face sessions (60 min), personalized feedback reports, private Facebook group to facilitate social support and engagement (reminders and notifications), Gymstick resistance band to facilitate home-based strength training, and finally a TEMPlate dinner disc to guide main meal portion size [76]. In contrast, the study conducted by West et al (2016) had an educational focus and comprised 8 sessions delivered weekly via electronic newsletters and a (private) Facebook group [72]. The intervention encouraged frequent self-weighing, regular PA, and healthy eating. Participants received a Wi-Fi enabled scale and a wearable PA tracker (Fitbit Zip) to facilitate self-monitoring and weight maintenance. Similarly, the *Tweeting to Health* intervention used a Twitter account to deliver education-based content. Participants also received a wearable PA tracker (Fitbit Zip) to facilitate self-monitoring [71].

#### Quality Assessment

According to the EPHPP quality assessment tool, overall, 19 out of the 24 included studies were rated as weak [57-59,61-72,75,77,78], 5 as moderate [60,73,74,76,79], and none as strong. A summary of the individual component ratings and overall quality ratings is provided in Multimedia Appendix 4. In terms of selection bias, no study reported representative sampling, with the majority using convenience sampling to recruit eligible participants from university or college-based settings. Participation rates were difficult to determine in most studies as details on consent throughout the recruitment, screening, and randomization stages were not clearly reported. Therefore, all 24 studies were classified as weak for *Component A*. With respect to study design, 92% (n=22) of the studies employed a CCT [63,65-68,70] or RCT [57-62,69,72-79] design. The 2 remaining studies used cohort designs: 1 employed a 1-group pretest-posttest design [64] and the other employed a 1-group posttest only design [71]. Consequently, 22 studies were rated as strong and 2 as moderate for *Component B*. In terms of confounders, 13 studies [57-63,69,72-75,78] reported no significant differences between intervention and comparison (control) groups at baseline; 3 [76,77,79] reported significant differences among groups but controlled for these in analyses; 3 [68,70] did not report any potential confounders; 3 [65-67] reported significant differences between groups at baseline but did not report whether these differences were controlled for; 2 [64,71] did not include a comparison group and therefore group differences were not applicable. Consequently, 16 studies [57-63,69,72-79] were rated as strong and 8 [62,65-68,70,74] as weak for *Component C*.

In terms of blinding, 16 studies [57-59,61,63,65-70,72,75,77-79] did not describe blinding of outcome assessors or participants. Owing to the recruitment methods employed and the nature of the interventions (ie, behavioral modification), participants in these studies were assessed as being aware of the study’s research question unless explicitly stated otherwise. Of the remaining studies, 2 studies [62,74] were double blinded; 1 [73] did not report blinding of outcome assessors but participants were reportedly blinded; 2 reported outcome assessors as blinded but participants as not [60,76]; blinding was not applicable in 2 studies [64,71]. Subsequently, 18 studies [57-59,61,63-72,75,77,78] were assessed as weak, 4 [60,73,76,79] as moderate, and 2 [62,74] as strong for *Component D*. With regard to data collection methods, 20 studies [57-63,65-69,72-79] reported some evidence of reliability (eg, Cronbach alpha) and validity (eg, reference to a validation study) for measures used to assess primary outcomes; 1 [64] reported measures to be valid but not reliable; 3 [70,71] did not report any evidence of the reliability and validity of the measures used. Consequently, 20 studies were assessed as strong [57-63,65-69,72-79], 1 as moderate [64], and 3 [70,71] as weak for *Component E*.

With respect to withdrawals and dropouts, 12 studies [57,58,61,65,66,68-70,72,77,78] reported the number of dropouts but not the reasons for this attrition; 9 [59,60,63,67,73-76,79] reported both the numbers and reasons (eg, medical reasons, life changes could no longer commit, no contact); 1 study [64] did not report numbers or reasons. For the 2 remaining studies, withdrawals and dropouts were unclear [62] and not applicable [71]. On the basis of study completion rates, 10 studies [59,60,65,67,72-76,79] were assessed as strong (80%-100% retention), 8 [61,63,66,68-70,77] as moderate (60%-79% retention), and 6 [57,58,62,64,71,78] as weak (<60% retention) for *Component F*.

## Discussion

### Principal Findings

This systematic review provides a comprehensive narrative evidence synthesis of eHealth weight management interventions targeting young adults, with a particular focus on (eHealth) intervention components and outcomes. A total of 24 studies were identified and included in the review. A majority were published from 2010 onward, conducted in developed countries, and used convenience sampling to recruit young adults from university- or college-based settings. There was large variation in the behavioral focus, intervention design and duration, sample size, and outcomes reported across the included studies. The variability across intervention outcomes highlights that additional research is warranted to extend our understanding of *what works, for whom, how, and when?* The following discussion provides further commentary on review findings, along with recommendations for future research.

### Intervention (Electronic Health) Components

Technology as a means to communicate content in eHealth interventions is often overlooked [80]. Frequently, technology is seen as a black box, a mere tool that has no effect or value and serves only as a vehicle to deliver intervention content [81]. However, recent research suggests that technology should be seen as a vital and inseparable aspect of interventions [82] and should be examined from a more holistic perspective [81,83]. With differences in persuasive technology elements and user interaction shown to be significant predictors of adherence [80], the design of *persuasive technology* should be an important consideration in the development of any eHealth intervention seeking sustained adherence [84]. Nonadherence is an issue that continues to plague the effectiveness of eHealth interventions [80,82,85], with many participants failing to sustain their use of the intervention in the desired way [81]. Given that nonoptimal exposure to an intervention has been shown to lessen intervention effect [86], examining technology and user interaction from a more holistic perspective is necessary for improving adherence and in turn the effectiveness of eHealth interventions.

In this review, the use of eHealth components and behavioral change strategies varied, with some studies only utilizing 1 technological function (eg, SMS text message or email) and others employing a range of internet- and mobile-enabled functions (eg, website, mobile phone apps, email, and SMS text message). Earlier studies (published 2006-2012) were generally more basic by design with the majority employing an e-learning-based approach to deliver a didactic education-focused weight management program, usually as part of a college or university-based course. With a focus on education and raising awareness, these interventions offered limited opportunities for participants to interact and actively engage with the technology, and as a result, exposure to intervention content was likely suboptimal. Interaction has been shown to be a significant predictor of adherence; therefore, eHealth interventions that fail to promote user interaction are unlikely to achieve the intended usage target [81]. Furthermore, research indicates that focusing on education (ie, knowledge shaping) alone is unlikely to achieve the level of behavior change necessary to address weight status [87,88]. Moreover, participants enrolled in the e-learning-based interventions were likely to be more motivated by the course credit on offer rather than learning new skills for healthy lifestyle adoption and weight maintenance. Later studies (published 2013-2017) became more sophisticated in their use of technology and associated behavioral change strategies, leveraging modern technological innovations. For example, using algorithms for content tailoring (eg, knowledge shaping), mobile phone apps and wearable devices for tracking behavior (eg, goal setting and self-monitoring) and relaying real-time feedback (eg, prompt review and reinforcement) to improve the capability, interactivity, and mobility of the intervention. Research has found eHealth interventions, which are enhanced by a range of features (eg, personalized e-feedback, chat rooms, and goal-setting and self-monitoring tools), support greater retention and usage of the intervention than standard (or basic) eHealth interventions [89]. It should be noted that 5 studies [59,60,65,67,76] also incorporated face-to-face (individual or group-based) sessions in 1 or more of the intervention (treatment) arms, further highlighting the limited evidence base for eHealth weight management interventions targeting young adults.

### Recommended Intervention Strategies Delivered Via Electronic Health

Although the evidence for successful eHealth weight management interventions targeting young adults (aged 18 to 35 years) was limited, common behavioral change strategies and techniques were able to be thematically identified, with an emphasis placed on the studies categorized as having positive or mixed weight-related outcomes. The 5 strategies identified included the following: self-regulation (goal setting and self-monitoring), tailored or personalized feedback, contact with an interventionist, social support, and behavioral prompts (nudges and reminders) and booster messages.

#### Self-Regulation (Via Goal Setting and Self-Monitoring)

Most weight management interventions promote goal setting along with some form of self-monitoring, usually recommending that participants should record details pertaining to their behavioral patterns (eg, dietary intake and PA) and weight (eg, BMI) and review tracking data in line with their goals or recommended guidelines to evaluate progress and identify where further changes are needed [90]. The premise of self-regulation for changing finely ingrained habits is that monitoring of one’s behavior will lead to self-evaluation of progress made toward previously set goals, with ensuing self-reinforcement following this evaluation. Thus, the process of changing habits requires well-developed self-regulatory skills [91,92]. Self-monitoring and goal setting are central to this process [93]. Self-monitoring requires deliberate attention to one’s own actions, as well as the conditions under which they occur, and their immediate and long-term effects [93]. Research indicates that self-monitoring of key behaviors has been associated with successful weight maintenance [92,94-96]. In particular, the use of technology for self-monitoring has been suggested as a way of lessening the burden of self-monitoring and enhancing adherence [97]. In this review, all studies reporting positive weight-related outcomes implemented some form of self-monitoring (eg, frequent self-weighing, monitoring PA, or dietary intake). For instance, in the HEYMAN intervention [76], participants received a Jawbone wearable PA tracker with an associated mobile phone app (UP app) to assist in goal setting and self-monitoring and a TEMPlate dinner disc to guide main meal portion size. The findings from this review suggest that improving self-regulation skills should be a central focus of future eHealth weight management interventions, particularly given young adults often lack such skills [24].

#### Tailored or Personalized Feedback

Tailoring has been shown to enhance the effectiveness of behavior change interventions, including eHealth-based interventions [94,95]. Tailoring involves gathering and assessing personal data to determine the most effective strategy to meet the specific needs of an individual [96]. Collecting data for tailoring intervention content enables personalized feedback, commands greater attention and is processed more deeply by the recipient and is perceived as more likable than a generic message [96,98]. With ready access to data provision and retrieval, the internet provides a powerful tool for tailoring interventions [96]. Interactive and responsive tailoring enhances the user’s experience with and understanding of intervention content [94,95]. Tailoring can range from simple Web-based assessments and feedback to highly sophisticated interventions that are completely customized [95]. Of the studies reporting tailoring in this review, most only employed simple tailoring based on either Web-based or in-person health assessments. For example, in the trial conducted by Bertz et al (2015), participants weighed themselves daily using Wi-Fi scales and immediately received an email containing their weight plotted over time with a horizontal reference line indicating their target weight [63]. A few studies employed more sophisticated levels of tailoring. The TXT2BFiT intervention [74] used a staging algorithm based on the Transtheoretical/Stages of Change Model to generate a personalized set of SMS text messages, which were tailored to whether the participant was in precontemplation, contemplation, preparation, action, or maintenance stages of change for each of the 4 behaviors addressed. More cognitive messages were included if a participant was in 1 of the early stages of change, and the messages were more behavioral if the participant was in the action or maintenance stages of change. We recommend that future studies experiment with more sophisticated methods of tailoring to empirically test which aspects of the tailored messages promote adherence and in turn enhance effectiveness in this context.

#### Contact With an Interventionist

Several studies included in this review incorporated in-person support from an interventionist. Human support has been shown to enhance the effectiveness of and adherence to eHealth interventions via accountability to a coach who is seen as trustworthy, benevolent, and having expertise [99]. However, intervention designs incorporating in-person support are resource intensive. A trained specialist is needed to deliver intervention content and monitor participants to ensure the correct treatment dose is received and the fidelity of the intervention is maintained. In addition, the facilities and equipment required to deliver the intervention must be procured. Furthermore, the effort that is required on the part of participants to commit to and attend in-person counseling sessions can create barriers (eg, cost of travel, lack of parking at venues, and limited availability) to full participation and adherence [100]. The high cost and inability of these interventions to reach diverse demographic and socioeconomic groups thwart large-scale deployment to the wider community [28]. More economical methods that may provide similar outcomes to face-to-face contact, while reducing the costs associated with intervention delivery, include coaching calls via telephone, email, chat forums, and SNSs [101]. For example, in the TXT2BFiT intervention [74], participants received 5 coaching calls focused on goal setting and a review from a dietician skilled in motivation interviewing. Similarly, in the CHOICES intervention [59], a study specific SNS facilitated participant engagement with peers and the intervention staff. Future research should consider the potential benefits and disadvantages of different communication mediums to deliver expert support at scale.

#### Social Support

Social support has been identified as an important factor in the provision of lifestyle-focused weight management interventions [102-104], including those supported by technology [105]. In particular, SNSs provide an ideal platform for facilitating social support with access to large existing (or new) networks of influencers [106]. The studies incorporating social support in this review typically facilitated peer support via online chat forums or SNSs. For example, in the CHOICES intervention conducted by Lytle et al (2017), a study-specific SNS was created to encourage discussion and interaction among participants [59]. Similarly, in the HEYMAN intervention, a combination of in-person (via group-based sessions) and Web-based social support (via a private Facebook group) was employed to facilitate interaction among participants [76]. Given that there is research to show that social contacts and normative beliefs influence weight status and intentions for weight control in young adults [106], mediums for delivering social support should be a key consideration in future research.

#### Behavioral Prompts (Nudges and Reminders) and Booster Messages

Maintenance of behavior change presents an ongoing challenge for behavior change research, with very little actually known about the process of behavioral maintenance [107]. The evidence supporting the use of behavioral nudges, reminders, and booster sessions for behavioral change and maintenance is mixed [108]. However, findings from this review indicate that booster emails, SMS text messages, and coaching calls may help promote behavioral maintenance over the longer term. For example, in the TXT2BFiT intervention [74], a low dose maintenance phase was implemented following the completion of the initial 12-week intervention. In this maintenance phase, participants received monthly SMS text messages and emails and 2 booster coaching calls at 5 and 8 months. Technology offers a feasible means of delivering strategies that promote behavioral maintenance; however, further research is needed to better understand the process of behavioral maintenance.

### Quality of Included Studies

A majority of studies included in this review were of weak methodological quality according to EPHPP quality assessment ratings. The main weaknesses identified were the following: a lack of studies employing representative sampling, not clearly reporting participation rates, not blinding assessors and participants to group allocation, and low completion rates. Future research should aim to address these issues to improve the methodological quality of the evidence for eHealth weight management interventions targeting young adults.

Representativeness in eHealth-based research is crucial for ensuring interventions are capable of reaching large, representative numbers of the target population, particularly those who are most in need of treatment [109]. According to Glasgow (2007), reach is a function of both participation rate and the representativeness of participants compared with nonparticipants based upon a set of key characteristics including race, ethnicity, socioeconomic status, computer experience, and health literacy [109]. Participant characteristics across the studies included in this review were similar, with the majority of participants recruited from large western universities or colleges using convenience sampling procedures. Consequently, the results obtained from these studies are limited to a very small, homogenous (ie, high socioeconomic status, education level, and health literacy level) subgroup of the target population, and they are unlikely to generalize to the larger target population, including those most in need (ie, socioeconomically disadvantaged groups, low education and health literacy, ethnic minorities, and rural and remote communities) [28]. The study conducted by Simons et al (2018) is the exception as investigators specifically recruited lower-educated working young adults. Although difficulties in recruiting young adults are acknowledged [110,111], to improve representativeness, future research should aim to employ probability sampling methods, maintain a careful record of recruitment strategies and results, and collect data on both participant and nonparticipant characteristics.

The design of most studies included in this review was rated as strong; however, very few reported blinding of outcome assessors and participants. As a consequence, findings from these studies were likely influenced by detection and reporting bias [55]. Importantly, the 2 studies [62,74] that were reportedly double blinded reported positive weight-related outcomes, and 2 out of the 3 studies [60,73,76] reporting some level of blinding (either outcome assessors or participants) reported positive weight-related outcomes. Therefore, where practical and feasible, future research should aim for double-blinded allocations.

Finally, intervention durations and completion rates varied significantly across studies (6 weeks-24 months for duration; 42%-98% for retention), which affects the veracity of study findings and the ability to compare outcomes. Coupled with the lack of studies reporting details on effective recruitment strategies and reasons for attrition, the current understandings on how best to recruit and engage young adults in weight management studies is limited [28,112,113]. To improve participation rates, retention, and resource allocation efficiency, future research should keep a careful record of recruitment strategies, participation rates, and reasons for attrition by following up with withdrawals and dropouts.

### Limitations

A number of limitations, many of which represent opportunities for future research, are acknowledged. First, the search parameters employed were specific to the review’s research aim, thereby limiting the number of studies identified. For example, grey literature, nonpeer-reviewed research, and studies not published in English were excluded. Future research may therefore extend this review by including grey literature, nonpeer-reviewed research, and studies not published in English. Moreover, all studies included in this review were conducted in developed countries; however, obesity is not isolated to the developed world [114]. Thus, extending the current review to include research from developing countries could provide valuable insight into the generalizability of study findings in different geographic contexts. Furthermore, future research may adopt the Patient, Problem or Population, Intervention, Comparison, Control or Comparator, and Outcomes framework to inform the search strategy and eligibility criteria and compare whether this approach yields the same or different results as in this review. Second, the highly complex nature of the interventions included in this review limited our ability to confidently isolate the active drivers of intervention outcomes [112]. Although some potential behavioral change strategies were thematically identified, definitive conclusions as to which intervention components were contributing most to outcomes (or lack thereof) were not able to be made. Future research should follow published guidelines on developing and evaluating complex interventions to permit critical appraisal [112], and research is called for to expand the evidence base. As the evidence base grows, we recommend that narrower age ranges should be set to extend understanding. Third, the EPHPP quality assessment tool, although deemed appropriate for the purposes of this review, is one of several tools that can be used to evaluate the quality of quantitative studies. As such, overall quality ratings should be interpreted with the specific characteristics of this tool in mind, given different assessment outcomes can arise from different tools [54].

Furthermore, obtaining representative samples and blinding participants to group allocation in interventions attempting to modify behavior(s) are not often practical or feasible. Assessment tools such as the EPHPP are principally concerned with *what should be* and this is seldom equivalent to *what works* in the field. Therefore, although the quality of the evidence in this review was generally assessed as weak, results should be interpreted tentatively. Sampling, study design, and retention rates will remain key determining factors of reliability and validity; however, further research attention should be directed toward the applicability, generalizability, and impact potential of studies. Given a large proportion of weight management interventions are delivered in-field, with varying budget amounts, expecting study designs to conform to the standards set within quality assessment tools arising from controlled clinical settings may not be realistic. As such, additional research is needed to better understand which metrics can be reliably applied within different research designs. For instance, future reviews may consider incorporating the Grading of Recommendations Assessment, Development and Evaluation [113] assessment tool to provide an overall judgement of the evidence base and in turn guide future practice. Future research may opt to narrow the scope of review to 1 specific behavior or study design to permit metaanalytic comparisons; however, the results of this review suggest such a narrow scope would significantly limit the number of eligible studies available for quantitative comparison at this point in time. Finally, we recommend that future studies publish a review protocol (researchprotocols.org) to establish an early scientific record, promote transparency, solicit early feedback, and enhance review methods and processes.

### Conclusions

The prevention of unhealthy weight gain in young adults provides a new target for reducing the rising prevalence of obesity, and it is one that could offer an effective transgenerational approach to obesity prevention. Consequently, there is a need to develop effective weight management programs that are capable of engaging a large number of young adults in healthy lifestyle adoption over the longer term. An eHealth-based approach offers potential, with young adults among the highest users of digital technologies. However, at present, there is limited high-quality, peer-reviewed evidence available. Future research must be directed toward improving the methodological quality of the evidence and establishing which specific elements of eHealth weight management interventions are most effective in achieving the desired outcomes, thereby answering the *for whom, how, and when* question.

## Appendix

Multimedia Appendix 1 Preferred Reporting Items for Systematic Reviews and Meta-Analyses checklist completed during the review.

Multimedia Appendix 2 Search strategy and database results.

Multimedia Appendix 3 Summary of individual study characteristics.

Multimedia Appendix 4 Summary of Effective Public Health Practice Project quality assessment results.

## Abbreviations

BMI: body mass index
CCT: controlled clinical trial
CCRB: Cochrane Collaboration Risk of Bias
CHOICES: Choosing Healthy Options in College Environments and Settings
eHealth: electronic health
EPHPP: Effective Public Health Practice Project
ICT: information and communication technology
PA: physical activity
PRISMA: Preferred Reporting Items for Systematic Reviews and Meta-analysis
RCT: randomized controlled trial
SMS: short message service
SNS: social network site
