# Supplementary material for: Weight Management in Young Adults: Systematic Review of Electronic Health Intervention Components and Outcomes
Source: J Med Internet Res. 2019 Feb 6;21(2):e10265. doi: 10.2196/10265 (PMC6381405; doi:10.2196/10265)
Supplement: Multimedia Appendix 1 [file jmir_v21i2e10265_app1.pdf]

Section/topic	#	Checklist item	Reported on page #
**TITLE**			
Title	1	Identify the report as a systematic review, meta-analysis, or both.	This study is explicitly identified as a *systematic review* on **p. 1**.
**ABSTRACT**			
Structured summary	2	Provide a structured summary including, as applicable: background; objectives; data sources; study eligibility criteria, participants, and interventions; study appraisal and synthesis methods; results; limitations; conclusions and implications of key findings; systematic review registration number.	A structured abstract is provided on **p. 1.**
**INTRODUCTION**			
Rationale	3	Describe the rationale for the review in the context of what is already known.	A rationale for this review is provided on **pp. 3– 5.**
Objectives	4	Provide an explicit statement of questions being addressed with reference to participants, interventions, comparisons, outcomes, and study design (PICOS).	Objectives of this review are outlined on **p. 5.**
**METHODS**			
Protocol and registration	5	Indicate if a review protocol exists, if and where it can be accessed (e.g., Web address), and, if available, provide registration information including registration number.	A published review protocol does not exist for this review. A recommendation is made regarding publishing review protocols on **p. 22.**
Eligibility criteria	6	Specify study characteristics (e.g., PICOS, length of follow-up) and report characteristics (e.g., years considered, language, publication status) used as criteria for eligibility, giving rationale.	Eligibility criteria are clearly outlined on **p. 7-8.**
Information sources	7	Describe all information sources (e.g., databases with dates of coverage, contact with study authors to identify additional studies) in the search and date last searched.	Information (data) sources used in this review are listed on **p. 6** and the full strategy is presented in **Multimedia Appendix 1.**
Search	8	Present full electronic search strategy for at least one database, including any limits used, such that it could be repeated.	The full electronic search strategy, including search results for each database, is provided in **Multimedia Appendix 1.**
Study selection	9	State the process for selecting studies (i.e., screening,	The study selection process is summarised in the PRISMA flowchart provided on **p. 10** (refer to

		eligibility, included in systematic review, and, if applicable, included in the meta-analysis).	**Figure 1).**
Data collection process	10	Describe method of data extraction from reports (e.g., piloted forms, independently, in duplicate) and any processes for obtaining and confirming data from investigators.	The data collection process employed in this review is outlined on **p. 8.**
Data items	11	List and define all variables for which data were sought (e.g., PICOS, funding sources) and any assumptions and simplifications made.	A list of data items extracted is provided on **p. 8,** with study characteristics summarised in **Multimedia Appendix 3.**
Risk of bias in individual studies	12	Describe methods used for assessing risk of bias of individual studies (including specification of whether this was done at the study or outcome level), and how this information is to be used in any data synthesis.	The EPHPP Quality Assessment Tool was used to assign an overall methodological quality rating for each include study. The tool assesses six components including: selection bias; study design; confounders; blinding; data collection methods; and withdrawals and dropouts. **Multimedia Appendix 4** presents a summary of the quality assessment results.
Summary measures	13	State the principal summary measures (e.g., risk ratio, difference in means).	N/A (see Item 1).
Synthesis of results	14	Describe the methods of handling data and combining results of studies, if done, including measures of consistency (e.g., I2) for each meta-analysis.	The evidence synthesis process is outlined on **p. 9.**
Risk of bias across studies	15	Specify any assessment of risk of bias that may affect the cumulative evidence (e.g., publication bias, selective reporting within studies).	N/A (see Item 12).
Additional analyses	16	Describe methods of additional analyses (e.g., sensitivity or subgroup analyses, meta-regression), if done, indicating which were pre-specified.	N/A (see Item 1 and 14).
**RESULTS**			
Study selection	17	Give numbers of studies screened, assessed for eligibility, and included in the review, with reasons for exclusions at each stage, ideally with a flow diagram.	The study selection process is summarised in **Figure 1** (**p. 10**).
Study characteristics	18	For each study, present characteristics for which data were extracted (e.g., study size, PICOS, follow-up period) and provide the citations.	Study characteristics are summarised on **p. 10** and presented in **Multimedia Appendix 3.**
Risk of bias within studies	19	Present data on risk of bias of each study and, if available, any outcome level assessment (see item 12).	Results of the EPHPP Quality Assessment are discussed on **pp. 13-15** and further summarised in **Multimedia Appendix 4.**
Results of individual studies	20	For all outcomes considered (benefits or harms), present, for each study: (a) simple summary data for each intervention	N/A (see Item 1 and 14).

		group (b) effect estimates and confidence intervals, ideally with a forest plot.	
Synthesis of results	21	Present results of each meta-analysis done, including confidence intervals and measures of consistency.	N/A (see Item 1 and 14).
Risk of bias across studies	22	Present results of any assessment of risk of bias across studies (see Item 15).	N/A (see Item 12).
Additional analysis	23	Give results of additional analyses, if done (e.g., sensitivity or subgroup analyses, meta-regression [see Item 16]).	N/A (see Item 1 and 14).
**DISCUSSION**			
Summary of evidence	24	Summarize the main findings including the strength of evidence for each main outcome; consider their relevance to key groups (e.g., healthcare providers, users, and policy makers).	Principal findings are summarised on **p. 15.** A discussion along with recommendations for future interventions is provided on **pp. 15 – 23.**
Limitations	25	Discuss limitations at study and outcome level (e.g., risk of bias), and at review-level (e.g., incomplete retrieval of identified research, reporting bias).	The strengths and limitations of the review are outlined on **pp. 21 – 22.**
Conclusions	26	Provide a general interpretation of the results in the context of other evidence, and implications for future research.	A general interpretation of review findings is provided on **p. 23.**
**FUNDING**			
	27	Describe sources of funding for the systematic review and other support (e.g., supply of data); role of funders for the systematic review.	No significant financial support was received that could have influenced the outcome of the review. No funding sources declared.

Checklist source: http://prisma-statement.org/documents/PRISMA%202009%20checklist.pdf
