# Supplementary material for: Weight Management in Young Adults: Systematic Review of Electronic Health Intervention Components and Outcomes
Source: J Med Internet Res. 2019 Feb 6;21(2):e10265. doi: 10.2196/10265 (PMC6381405; doi:10.2196/10265)
Supplement: Multimedia Appendix 2 [file jmir_v21i2e10265_app2.pdf]

## *Search terms*

| Search | Query |
|---|---|
| #1 | "digital health" OR "online intervention" OR "internet based" OR "web based" OR "electronic health" OR eHealth OR "mobile health" OR mHealth OR "electronic mail" OR email OR "text messaging" OR telemedicine OR telehealth |
| #2 | overweight OR obesity OR "weight management" OR "weight control" OR "weight gain" OR "weight gain prevention" OR "weight loss" OR diet* OR nutrition OR "physical activity" OR exercise |
| #3 | intervention* OR "randomi?ed controlled trial" OR evaluation OR trial* OR campaign* OR program* OR study |
| #4 | "young adult*" OR "college student*" OR "university student*" OR "emerging adult*" OR "young people" |
| #5 | #1 AND #2 AND #3 AND #4 |
| Limits | English language, peer reviewed, and human |

## *Databases and search results*

| Database | Search selection | Hits from search |
|---|---|---|
| CINAHL (via EBSCO*host*) | AB, TI, SU | 163 |
| Cochrane Library | AB, TI, KW | 282 |
| EBSCO (all databases) | AB, TI, SU | 195 |
| Embase | AB, TI | 248 |
| Emerald (full-text) | AB, TI, KW | 0 |
| ERIC (via ProQuest) | AB, TI, SU | 37 |
| MEDLINE (via EBSCO*host*) | AB, TI, SU | 237 |
| Ovid (all databases) | AB, KW, SU | 321 |
| ProQuest (all databases) | AB, TI, SU | 126 |
| PsycINFO (via Ovid) | AB, KW, SU | 120 |
| PubMed | AB, TI | 64 |
| Science Direct (expert search) | TITLE-ABSTR-KEY | 4 |
| Scopus | TI, AB, KW | 1038 |
| Web of Science (Core collection) | TS, TI | 445 |
| Total | | 3280 |
| Total after duplicates removed | | 1301 |

Note: AB = abstract, KW = keywords, SU = subject terms/headings, TI = title/document title, TS = topic
