# Supplementary material for: Weight Management in Young Adults: Systematic Review of Electronic Health Intervention Components and Outcomes
Source: J Med Internet Res. 2019 Feb 6;21(2):e10265. doi: 10.2196/10265 (PMC6381405; doi:10.2196/10265)
Supplement: Multimedia Appendix 3 [file jmir_v21i2e10265_app3.pdf]

Author/year	Country	Design	*N*	Retention	Objective	Intervention (I)/comparator (C)	eHealth component(s)	Key strategies	Duration	Measure	Outcome(s)	Effect
**Web-based**												
Levitsky et al., 2006a	USA	CCT	32	81%	Weight management (self-weighing)	I: daily self-weighing emailed to an interventionist, with a linear function emailed back.<br>C: no intervention.	Email	- Self-monitoring<br>- Contact with interventionist (daily)	12 wks.	Weight	Controls gained 3.17 plus-minus 0.51 kg, (*P* < 0.01), whereas weight gain of the experimental group was 0.17 plus-minus 0.99kg.	+
Levitsky et al., 2006b	USA	CCT	41	78%	Weight management (self-weighing)	I: daily self-weighing emailed to an interventionist, with recommended caloric feedback emailed back.<br>C: no intervention.	Email	- Self-monitoring<br>- Contact with interventionist (daily)	12 wks.	Weight	Controls gained 2.07 plus-minus 0.65 kg (P < 0.01), whereas weight gain of the experimental group was -0.827 plus-minus 0.56 kg.	+
Gow et al., 2010	USA	RCT	159	69%[a, b]	Weight management (diet & PA)	I[1]: 6 intensive sessions delivered via Blackboard focused on the factors involved in healthy weight maintenance.<br>I[2]: weekly self-weighing and recording on Blackboard, with recommended caloric change emailed to participants.<br>I[3]: received both I's.<br>C: no intervention.	eLearning website<br>Email	- Education/awareness raising<br>- Self-monitoring<br>- Social support<br>- Skill development	6 wks.	BMI	CI group had significantly lower BMI scores (24.13, SE =.09) than the control group (M = 24.56, SE = .09, *P* < .05); however, the II group and FI group did not significantly differ on BMI compared to the control group (*P* > .05).	+/-
Wadsworth & Hallam, 2010	USA	CCT	91	78%	Weight management (PA)	I: 6 weekly emails directed participants to webpages that targeted SCT variables. Each webpage ended with a short survey. Participants had access to an e-counsellor and were sent 4 monthly booster emails.<br>C: no intervention.	eLearning website<br>Email	- Goal setting<br>- Self-monitoring<br>- Social support<br>- Reinforcement<br>- Relapse prevention<br>- (Realistic) expectation setting	6 wks.	BMI<br>BF	No significant differences in BMI or BF (%). Significant (*P* < .05) difference between groups for frequency of moderate PA at 6 wks. (not maintained at 6 mo.).	NIL
Dennis et al., 2012	USA	CCT	45	87%	Weight management (diet & PA)	I[1]: online modules and bi-weekly in-class sessions focused on outcome expectations and self-efficacy.<br>I[2]: I[1] plus explicit self-regulation features utilising an online tracking program.	eLearning website	- Outcome expectations<br>- Self-efficacy<br>- Self-monitoring	14 wks.	Weight BMI<br>BF	Weight increased over the 14-wk. period, but there were no group differences. BF (%) increased in SCTSR but not SCT (mean difference: SCTSR, +1.63 plus-minus 0.52%; SCT, −0.25 plus-minus 0.45%; *P* < .01). No group differences for BMI.	+/-
Greene et al., 2012	USA	CCT	1689	67%	Weight management (diet & PA)	I: weekly online lessons and focused on improving attitudes, self-efficacy, and behaviours to facilitate weight management. Individualised profile pages displaying assessment results, graph of weekly goals and self-reported behaviour compared to recommendations.<br>C: access to a profile page with data from each assessment and an explanation of results. No further intervention received (waitlist control).	eLearning website	- Knowledge shaping<br>- Goal setting<br>- Self-monitoring<br>- Personalised feedback (comparative)	10 wks.	Weight<br>BMI	Both groups increased weight, height, BMI, and waist circumference over the 15-mo. Intervention group had significantly (*P* < .001) higher FV intake and PA participation than the control group.	NIL
LaChausse et al., 2012	USA	CCT	320	98%	Weight management (diet & PA)	I[1]: online education program comprised of 4 Rate Myself assessments, 3 information links, and 4 main learning modules. MSB-N students were required to spend at least 2hrs per week (over 12 wks.) on the course (MSB-N).<br>I[2]: met weekly for 2 hrs over 12-wks. (on-campus intervention).<br>C: no intervention.	eLearning website	- Knowledge shaping<br>- Personalised feedback	12 wks.	BMI	No significant change in BMI among participants in intervention groups. Significant (p < .001) increase in FV intake observed among MSB-N participants.	NIL
Harvey-Berino et al., 2012	USA	Cohort study[c]	336	98%	Weight management (diet & PA)	I: divided into two groups (healthy weight & overweight/obese) based on BMI and attended closed group meetings (1hr/wk.) in an online synchronous chat led by an interventionist. Additional web-based	eLearning website	- Knowledge shaping<br>- Goal setting	12 wks.	BMI	Healthy weight students wanting to lose weight or achieve a healthy lifestyle lost an average of 3.2 plus-minus 4.1 lbs and 1.2 plus-minus 2.6 lbs (*P* < .001), respectively.	+

Author/year	Country	Design	*N*	Retention	Objective	Intervention (I)/comparator (C)	eHealth component(s)	Key strategies	Duration	Measure	Outcome(s)	Effect
						resources (e.g. journal, bulletin board, recipes, and BMI calculator) were provided. To pass students had to attend ≥10 meetings and complete ≥10 journals.<br>C: N/A	Online synchronous chat	- Contact with interventionist<br>- Self-monitoring<br>- Personalised feedback<br>- Social support Problem solving<br>- Stimulus control<br>- Relapse prevention<br>- Assertive skills			Overweight/obese students wanting to lose weight or achieve a healthy lifestyle lost an average of 6.1 plus-minus 5.8 lbs (*P* < .001) and 4.5 plus-minus 7.2 lbs (*P* < .01), respectively.[c]	
Bertz et al., 2015	USA	CCT	167	78%<br>ITT.	Weight management (self-weighing)	I: CTM (caloric titration method) participants were given a Wi-Fi scale and asked to weigh themselves daily. An emailed graph containing weight plotted over time with a horizontal reference line indicating the target weight was sent immediately after weighing.<br>C: Wi-Fi scale to weigh themselves on 3 days during 1 wk. at baseline, 6 mo. and 1 year. No data or feedback received.	Email<br>Wi-Fi scale	- Self-monitoring<br>- Personalised feedback	12 mo.	Weight<br>BMI	Control group had gained 1.1 plus-minus 4.4 kg at 12 mo. whereas the CTM group lost 0.5 plus-minus 3.7 kg (*F*53.39, *P* = 0.035). The difference in weight change between the two groups at 12 mo. was significant (*P* = 0.004). 95% of CTM participants weighed ≥ 3 times/wk., compared to 15% in the control group (*P* < 0.001).	+
Cameron et al., 2015[d]	UK	RCT	2621	42%<br>ITT.	Healthy lifestyle (diet, PA, responsible drinking & not smoking)	I: online health behaviour intervention delivered via a website and comprising a self-affirmation manipulation, 4 short modules on the 4 health behaviours (with theory-based messages and planning exercises), and access to additional health messages and links.<br>C: no intervention.	eLearning website	- Knowledge shaping<br>- Self-affirmation<br>- Action planning<br>- Self-monitoring	6 mo.	BMI	No significant differences between groups on any of the primary outcomes, or BMI, at follow-up (6 mo.).	NIL
Nikolaou et al., 2015	USA	RCT	20975	83%	Weight management (diet & PA)	I1: followed the rational model and addressed unwanted weight gain and obesity overtly (NTICVI).<br>I²: followed the stealth model and addressed unwanted weight gain and obesity covertly by raising discussion around social and political movements which are associated with more, or less, healthful diets and lifestyles (GDI).<br>Both I's were delivered in weekly instalments using the Moodle learning platform. Weekly reminder emails were sent informing participants of the topic for the week. Mailboxes were created for each group to allow communication between participants and the administrator.<br>C: no intervention.	eLearning website<br>Electronic forums/mail	- Knowledge shaping<br>- Reminders (prompts)<br>- Social support (+ contact with administrator)	19 wks.	BMI	Weight changes were significantly (*P* < 0.001) different between groups. Control: 2.0 kg (95% CI = 1.5, 2.3 kg); NTICVI: 1.0 kg (95% CI = -1.3, -0.5kg); GDI: -1.35 kg (95% CI = -1.4, -0.7) at 40 wks.	+
Schweitzer et al., 2016	USA	RCT	148	72%<br>ITT.	Weight management (diet & PA)	I: Modified eHealth intervention (NutritionQuest). Participants were asked to select a goal related to fat and sugar intake, FV intake, or PA. This goal formed the focus of weekly email messages offering tips for achieving the goal and weblinks to participants' personal accounts located within the intervention website where educational info. and feedback on progress were available.<br>C: received weekly info. related to non-diet, non-exercise health topics.	eLearning website<br>Email	- Knowledge shaping<br>- Goal setting<br>- Feedback	24 wks.	Weight<br>BMI	No significant differences between groups on any weight-related outcomes.	NIL
Wing et al., 2016	USA	RCT	599	87%	Weight management (diet, PA & self-weighing)	Interventions began with 10x F2F meetings over 4 mo. and were delivered online thereafter. Online refresher campaigns (2x 4 wks.) were offered each year. Participants were sent quarterly newsletters and personalised feedback reports.<br>I¹: (small changes) instructed to self-weigh daily and submit via the study website, text message, or email.	eLearning website<br>Email	- Knowledge shaping<br>- Personalised feedback<br>- Self-monitoring<br>- Graded tasks	24 mo.	Weight<br>BMI	Mean weight changes averaged across follow-up were: 0.26 plus-minus 0.22, -0.56 plus-minus 0.22, and -2.37 plus-minus 0.22 kg in the control, small-changes, and large-changes groups, respectively. All 3 pairwise comparisons were significant with less weight gain (and some weight loss) in	+

Author/year	Country	Design	*N*	Retention	Objective	Intervention (I)/comparator (C)	eHealth component(s)	Key strategies	Duration	Measure	Outcome(s)	Effect
						Received monthly feedback via email on their weight and were instructed to make small daily changes to diet and PA.<br>I^2: (large changes) followed the same protocol as the small changes group but were focused on making large changes. Participants were prescribed a calorie deficit during first 8 wks. and encouraged to increase PA.<br>C: minimal intervention - attended a F2F meeting and received quarterly newsletters and feedback reports.					the small-changes (P = .02) and large changes (P < .001) groups relative to control, and in the large changes relative to the small-changes group (P < .001). [f]	
Lytle et al., 2017	USA	RCT	441	83%	Weight management (diet, PA, sleep & stress)	I: academic course (online, F2F, or hybrid) focused on healthy weight management. Participants set goals for 10 unique lifestyle behaviours and weight. A study-specific SNS was used to encourage self-monitoring, goal setting, and interaction. The site included a discussion forum for students to engage with peers and intervention staff. Points for participation were provided and could be redeemed for a variety of wellness-related products. Periodic encouragement via email was also offered.<br>C: no intervention.	eLearning website<br>SNS	- Knowledge shaping<br>- Goal setting<br>- Self-monitoring<br>- Social support<br>- Contact with interventionist<br>- Reinforcement (rewards)	24 mo.	Weight<br>BMI<br>BF<br>Waist circumference	No statistically significant difference in weight, BMI, BF (%) or waist circumference between groups at 24 mo. Post-hoc analysis found the prevalence of overweight or obesity was significantly lower in the intervention condition compared with the control group at 24 mo. (net difference, 8.3%; P = 0.049). Intervention participants who were overweight or obese at baseline were more than 3x as likely to transition to a healthy weight by the end of the trial as compared with controls (P = 0.02).	+/-
**Mobile-based**												
Muñoz et al., 2014	USA	RCT	201	57%	Weight management (PA)	I: received pedometer with orientation and brief text messages (2-3 times/wk.) with suggestions for implementing healthy behaviours.<br>C: received no text communication.	Wearable tracking device<br>Text messages	- Self-monitoring<br>- Behavioural nudges (prompts)	16 wks.	Weight<br>BMI	Weight and BMI did not differ between groups at 16 wks.	NIL
Kerr et al., 2016	AUS	RCT	247	89%	Weight management (diet)	I^1: following baseline dietary analysis, tailored dietary feedback text messages were constructed for FV, EDNP food, and SSB (F+TI). In addition, participants received weekly text messages for 6 mo. (32 total).<br>I^2: Received 2x tailored dietary feedback messages only (FOI).<br>C: no intervention.	Text messages	- Personalised feedback<br>- Reinforcement (reminders)	6 mo.	Weight<br>BMI	FOI group significantly reduced weight (-1.7 [CI = -3.2, -0.3], P = .02) and BMI (-0.6 [CI = -1.1, -0.1], P = .02] compared with controls. No significant changes observed for the F+TI group.	+/-
Simons et al., 2018	Belgium	RCT	130	92%	Healthy lifestyle (PA)	I: Active Coach app combined with Fitbit activity tracker. Personal goals, practical tips, and educational facts were provided.<br>C: print-based generic PA information.	Application<br>Wearable tracking device	- Goal setting<br>- Self-monitoring<br>- Shaping knowledge	9 wks.	BMI	No differences in BMI reported. No significant intervention effects found for objectively measured PA, self-reported PA, and self-reported psychosocial variables (all P>.05).	NIL
**Multi-component**												
Hebden et al., 2014	AUS	RCT	51	90%	Weight management (diet & PA)	I: mHealth program including 2x tailored text messages (stage of change) and 2x emails/wk., smartphone app, and an internet forum for interaction with interventionist and other participants. At baseline, participants selected 2 behaviours (PA & sedentary behaviour, FV intake, takeaway meals or SSB intake) to work on during the program.<br>C: received a 10-page booklet which included national PA guidelines and instructions for a low-GI, high-protein diet. No further intervention was received.	Text messages<br>Emails<br>Smartphone application<br>Internet forum	- Behavioural nudges (prompt)<br>- Self-monitoring<br>- Tailored advice<br>- Social support<br>- Contact with interventionist	12 wks.	Weight<br>BMI	Post-intervention changes were not significantly *different* from controls. Intervention group decreased weight (-1.6 plus-minus 2.6 kg, P = 0.004) and BMI (-0.58 plus-minus 0.90 kg/m^2, P = 0.003); increased light intensity PA (34.2 plus-minus 35.1 min day^{-1}, P = 0.006), increased vegetable intake (1.0 median serving day^{-1}, P = 0.033), and decreased SSB intake (-355 plus-minus 836 mL week^{-1}, P = 0.040).	NIL

Author/year	Country	Design	*N*	Retention	Objective	Intervention (I)/comparator (C)	eHealth component(s)	Key strategies	Duration	Measure	Outcome(s)	Effect
Epton et al., 2014	UK	RCT	1445	63%	Healthy lifestyle (diet, PA, responsible drinking & not smoking)	I: online health behaviour intervention delivered via a website. Included a self-affirmation task, theory-based messages (text, videos & links), and an implementation intention planner. An android smartphone app was also available.<br>C: no intervention.	eLearning website<br>Smartphone application	- Knowledge shaping<br>- Self-affirmation<br>- Action planning<br>- Self-monitoring	12 mo.	BMI	No significant intervention effects on most primary outcomes (PA, alcohol or FV intake) or on BMI, at follow-up. Significant effect on smoking status at follow-up with fewer smokers in the intervention group (8.7%) than in the control group (13.0%; Odds ratio = 1.92, *P* = .010).	NIL
Kattelmann et al., 2014	USA	RCT	1639	59%	Weight management (diet, PA & stress)	I: website (incl. 21 mini-educational lessons) and email messages (4 nudges/wk.) focused on healthy weight management via a non-diet approach. Participants required to visit the website weekly, set goals, and track progress via a personalised graph. During the follow-up phase, nudges were reduced to 4/mo.<br>C: waitlist control	SNS<br>Text messages	- Knowledge shaping<br>- Goal setting<br>- Personalised feedback<br>- Behavioural nudges (prompt)	10 wks.	Weight<br>BMI<br>Waist circumference	No differences between groups on any weight-related measures. Improvements in FV intake (*P* < .001), vigorous PA in females (*P* < .05), fat intake (*P* < .05), self-instruction (*P* < .001), and regulation (*P* < .05) for mealtime behaviour, and sleep (*P* < .05) at 10 wks. (not maintained).	NIL
Allman-Farinelli et al., 2016	AUS	RCT	250	81%	Weight management (diet & PA)	I: mHealth program including 5 coaching calls conducted by a dietician skilled in MI and centred on goal setting and review; personalised text messages (8 texts/wk.) tailored to stage of change; weekly emails (12 total) which repeated the info. sent in the text messages; 18-page diet and nutrition booklet with PA guidelines; a website (incl. weight tracker, printable charts, PA planner, blog); and 4 designer smartphone apps for education and self-monitoring. Following program completion, participants received a low dose maintenance intervention which consisted of monthly text messages and emails, 2 booster coaching calls at 5 and 8 mo., and continued access to the website.<br>C: minimal intervention consisting of 4 text messages over 12 wks., a 2-page handout based on dietary and PA guidelines, access to a website containing participant info. sheet and 2-page handout. No further intervention received.	Telephone counselling (coaching calls)<br>Text messages<br>Email<br>Website<br>Smartphone applications	- Knowledge shaping<br>- Goal setting<br>- Prompt reviews<br>- Behavioural nudges (reminders)<br>- Self-monitoring<br>- Action planning<br>- Booster sessions	12 wks.	Weight<br>BMI	Intervention participants weighed 3.7 kg (95% CI = -6.1, −1.3, *P* = .003) and 4.3 kg (95% CI = -6.9, -1.8, *P* = .001) less than controls at 12 wks. and 9 mo., respectively. No differences in PA were found but all diet behaviours showed that the intervention group had significantly greater odds of meeting dietary recommendations than controls at 9 mo.	+
West et al., 2016	USA	RCT	58	97%	Weight management (diet, PA, & self-weighing)	I: 8-session healthy weight management intervention (HW) focused on frequent self-weighing, regular PA, and healthy eating. Participants received a Wi-Fi enabled scale and an electronic PA tracker (Fitbit Zip) to facilitate weight regulation. Content was delivered via weekly electronic newsletters and a (private) Facebook group.<br>C: Matched with HW intervention but focused on human papillomavirus (HPV) vaccination awareness.	eNewsletter<br>SNS<br>Wi-Fi scale<br>Wearable tracking device	- Self-monitoring<br>- Goal setting<br>- Graded tasks<br>- Social support<br>- Knowledge shaping<br>- Barrier identification<br>- Model/demonstrate	9 wks.	Weight<br>BMI	Both groups remained fairly weight stable over the 9 wks. (HW: -0.48 plus-minus 1.9 kg; control: -0.45 plus-minus 1.4 kg), with no significant Group × Time interaction (*P* = .94). The HW group significantly increased total number (2.1 plus-minus 4.5) of weight control strategies (self-weighing and graphing weight, cutting out snacking, reducing calorie, carb, and fat intake, and PA) compared to the control group (-1.1 plus-minus 3.4; *P* =.003).	NIL
Ashton et al., 2017	AUS	RCT	50	94%	Healthy lifestyle (not smoking, nutrition, alcohol, PA & obesity)	I: lifestyle program focused on SNAPO-related behaviours and consisting of a website (resource library), Jawbone™ wearable PA tracker with associated app, 1 hr weekly F2F sessions (11x group & 1x individual), personalised reports, private Facebook group to facilitate interaction with other participants and intervention staff, a Gymstick™ for home-based exercise, and a TEMPlate™ dinner disc to guide main meal portion size.<br>C: waitlist control.	Website<br>Wearable tracking device<br>Application<br>SNS	- Knowledge shaping<br>- Barrier identification<br>- Goal setting<br>- Outcome expectations<br>- Self-monitoring<br>- Graded tasks<br>- Skill development<br>- Personalised feedback<br>- Contact with interventionist<br>- Social support	3 mo.	Weight<br>BMI<br>Fat mass<br>Waist circumference	Significant (p < .05 or .001) intervention effects for weight (*d* = 0.63), weight loss (*d* = 0.67), BMI (*d* = 0.81), fat mass (*d* = 0.67), and waist circumference (*d* = 0.89) when compared with controls. Significant improvements found for daily vegetable servings (*d* = 0.62), EDNP foods (*d* = 0.73), moderate and vigorous PA (*d* = 0.58).	+

Author/year	Country	Design	*N*	Retention	Objective	Intervention (I)/comparator (C)	eHealth component(s)	Key strategies	Duration	Measure	Outcome(s)	Effect
Chung et al., 2017	USA	Cohort	12	N/G	Healthy lifestyle (diet & PA)	I: Received a Fitbit Zip wearable device with associated smartphone/desktop app with real-time data, and private Twitter and Fitbit accounts. Participants received Tweets that focused on increasing PA, increasing FV intake, and decreasing SSB intake. Personalised feedback was provided in the second month, and individual vs. group challenges were created as an added incentive.	Wearable tracking device<br>Application<br>SNS	- Self-monitoring<br>- Social support<br>- Personalised feedback<br>- Incentive (social reward)	2 mo.	Weight<br>BMI<br>BF	OW participants lost between 1-5 pounds and 3.9% to 10.6% BF vs. 0.2-7 pounds and 0.5% to 13.5% among HW participants. No tests of significance were conducted[g]	NIL

Note: + = positive outcome; +/- = mixed outcome; NIL = no significant change. Effect is based on reported weight-related outcomes for each – no comparison is made between study types.
N/G = not given; N/A = not applicable; data reported as mean plus-minus standard deviation, unless otherwise stated.
[a]Analyses of follow-up data were not conducted owing to high attrition (89%).
[b]Retention highest in CI group (79.5%), lowest in II and FI groups (56.1%, and 59%, respectively).
[C]Cohort study – one group pretest-posttest design (no comparison group)
[d]Repeat RCT of Epton et al., (2014).
[e]Based on self-reported baseline and follow-up body weight
[f]Some participants were expected to also complete years 3 and 4
[g]Single arm intervention – owing to the small sample size, tests of difference between the groups were not examined.
Abbreviations: BF (body fat); BMI (Body mass index); CCT (controlled clinical trial); EDNP (energy-dense, nutrient poor); FV (fruit and vegetable); ITT (intention-to-treat); PA (physical activity), RCT (randomised controlled trial); SCT (Social Cognitive Theory); SMS (short message service); SSB (sugar-sweetened beverage)
