# Supplementary material for: Weight Management in Young Adults: Systematic Review of Electronic Health Intervention Components and Outcomes
Source: J Med Internet Res. 2019 Feb 6;21(2):e10265. doi: 10.2196/10265 (PMC6381405; doi:10.2196/10265)
Supplement: Multimedia Appendix 4 [file jmir_v21i2e10265_app4.pdf]

Study	A.	B.	C.	D.	E.	F.	Global Rating
Levitsky et al., 2006a	*	***	*	*	*	**	*
Levitsky et al., 2006b	*	***	*	*	*	**	*
Gow et al., 2010	*	***	***	*	***	**	*
Wadsworth & Hallam, 2010	*	***	*	*	***	**	*
Dennis et al., 2012	*	***	*	*	***	***	*
Greene et al., 2012	*	***	*	*	***	**	*
LaChausse et al., 2012	*	***	*	*	***	***	*
Harvey-Berino et al., 2012	*	**	–	*	**	*	*
Kattelmann et al., 2014	*	***	***	*	***	*	*
Muñoz et al., 2014	*	***	***	*	***	*	*
Hebden et al., 2014	*	***	***	**	***	***	**
Epton et al., 2014	*	***	***	*	***	**	*
Bertz et al., 2015	*	***	***	*	***	**	*
Cameron et al., 2015	*	***	***	*	***	*	*
Nikolaou et al., 2015	*	***	***	***	***	*	*
Allman-Farinelli et al., 2016	*	***	***	***	***	***	**
Schweitzer et al., 2016	*	***	***	*	***	**	*
Wing et al., 2016	*	***	***	**	***	***	**
Kerr et al., 2016	*	***	***	*	***	***	*
West et al., 2016	*	***	***	*	***	***	*
Lytle et al., 2017	*	***	***	*	***	***	*
Ashton et al., 2017	*	***	***	**	***	***	**
Chung et al., 2017[a]	*	**	–	*	*	*	*
Simons et al., 2018	*	***	***	**	***	***	**

Note: * = weak; ** = moderate; *** = strong. N/A = not applicable; – = unable/inappropriate to score

Components: A. Selection Bias; B. Study Design; C. Confounders; D. Blinding; E. Data Collection Methods; F. Withdrawals and Drop-outs

[a]Cohort (one group pre + post) design, therefore between group differences are not applicable
